# A Novel Automatic Approach for Calculation of the Specific Binding Ratio in [I-123]FP-CIT SPECT

**DOI:** 10.3390/diagnostics10050289

**Published:** 2020-05-09

**Authors:** Mahmudur G. M. Rahman, Muhammad M. Islam, Tetsuya Tsujikawa, Hidehiko Okazawa

**Affiliations:** 1 Biomedical Imaging Research Center, University of Fukui, Eiheiji-cho 910-1193, Japan; dtuhin28@yahoo.com (M.G.M.R.); mmi@bme.kuet.ac.bd (M.M.I.); awaji@u-fukui.ac.jp (T.T.); 2 Department of Biomedical Engineering, Khulna University of Engineering & Technology, Khulna 9203, Bangladesh

**Keywords:** dopamine transporter, SPECT, ioflupane, specific binding ratio, automatic quantification, reproducibility

## Abstract

A fully automatic method for specific binding ratio (SBR) calculation in [^123^I]ioflupane single-photon emission computed tomography (SPECT) studies was proposed by creating volumes of interest of the striatum (VOI_st_) and reference region (VOI_ref_) without manual handling to avoid operator-induced variability. The study involved 105 patients (72 ± 10 years) suspected of parkinsonian syndrome (PS) who underwent [^123^I]ioflupane SPECT. The 200 images from our previous study were used for evaluation and validation of the new program. All patients were classified into PS and non-PS groups according to the results of clinical follow-up. A trapezoidal volume of interest (VOI_t_) containing all striatal intensive counts was created automatically, followed by VOI_st_ setting using the previous method. SBR values were calculated from the mean values of VOI_st_ and VOI_ref_ determined by the whole brain outside of VOI_t_. The low count voxels in the VOI_ref_ were excluded using an appropriate threshold. The SBR values from the new method were compared with the previous semi-automatic method and the Tossici–Bolt (TB) method. The SBRs from the semi- and fully automatic methods showed a good linear correlation (*r* > 0.98). The areas under the curves (AUCs) of receiver operating characteristic analysis showed no significant difference between the two methods for both our previous (AUC > 0.99) and new (AUC > 0.95) data. The diagnostic accuracy of the two methods showed similar results (>92%), and both were better than the TB method. The proposed method successfully created the automatic VOIs and calculated SBR rapidly (9 ± 1 s/patient), avoiding operator-induced variability and providing objective SBR results.

## 1. Introduction

Delineation of degenerative changes in the nigrostriatal dopaminergic neurons is a useful pathologic biomarker for differentiation of movement disorders [1]. Decline of dopamine transporter (DaT) expression was used for evaluation of the striatal neurodegenerative status in cases of parkinsonian syndrome (PS) or dementia with Lewy body (DLB). [^123^I]ioflupane (FP-CIT), the most common DaT ligand for single-photon emission computed tomography (SPECT), is usually used for evaluation of this degenerative alteration [2,3,4,5,6]. A quantitative assessment along with visual observation improves the diagnostic accuracy of DaT-SPECT imaging [7,8,9,10,11]. The specific binding ratio (SBR) calculated from the ratio of the striatal specific to non-specific binding in the brain is now commonly used as a quantitative index of parkinsonian syndrome. In order to calculate the SBR accurately, the volume of interest of the striatum (VOI_st_) and the reference region (VOI_ref_) should be created appropriately because SBR values are determined by the count–concentration ratio of these two regions. Since the manual method often causes operator-induced variability in quantification, several automatic and semi-automatic methods were proposed to acquire better reproducibility and diagnostic accuracy [12,13,14,15,16,17,18,19].

The semi-automatic method may not avoid the possibility of operator-induced variability during manual drawing of the VOI_ref_ on the occipital region, although it showed no significant SBR difference between two operators in a previous study [18]. To obtain more stable and reliable results, Buchert et al. applied a fixed percentage threshold of the maximum count for the reference region including only the gray matter in the VOI_ref_ [19], because distribution volumes of the tracer may differ between the white and gray matter [20]. Recently, Mizumura et al. calculated SBR using VOIs excluding voxels containing cerebrospinal fluid (CSF) [21] in a modification of the Tossici–Bolt (TB) method [22].

In the present study, a fully automatic method was proposed to avoid operator-induced variability that should be avoided for patient follow-up, as well as in multicenter studies. The whole-brain VOI_ref_, except for the striatal region, was applied to estimate reliable non-specific counts of the reference region after excluding the voxels containing CSF.

## 2. Materials and Methods

### 2.1. Patients

One hundred and five patients (52 males, 53 females, 72 ± 10 years), admitted to the Department of Neurology or Psychiatry, University of Fukui Hospital, were included in the present study. All patients were divided into two groups, either PS (*N* = 60) or non-PS (*N* = 45), after [^123^I]FP-CIT SPECT study and clinical follow-up for more than six months. We used data from 200 patients in our previous study to validate the new program and to determine an appropriate threshold value for elimination of the low count voxels from the VOI_ref_. The previous data were also used for comparison of the diagnostic performance with that of the new patient groups. The PS group included Parkinson’s disease, multiple system atrophy, progressive supranuclear palsy, corticobasal degeneration, and DLB. Patients with depressive illness, drug-induced parkinsonism, normal pressure hydrocephalus, and vascular parkinsonism were classified into the non-PS group. The study was designed as a retrospective cohort study aiming to improve the reproducibility and operability of SBR calculation in consecutive [^123^I]FP-CIT SPECT scans. The study protocol was approved (25. Feb. 2017) by the Ethics Committee, University of Fukui Hospital (#20170225).

### 2.2. SPECT Imaging

[^123^I]FP-CIT SPECT was performed 3–4 h after administration of about 170 MBq of tracer in the morning (167 MBq at noon) using a dual-head SPECT/CT scanner (Symbia T2, Siemens, Erlargen, Germany) and low- and medium-energy general-purpose collimators. The imaging parameters were the same as in the previous study [18]: 159 keV photo-peak and ±10% energy window, 128 × 128 matrix with 2.2 × 2.2 × 2.2 mm^3^ voxel size, zoom factor of 1.5. For triple-energy window scatter correction (SC), two additional 7% energy sub-windows were used on the upper and lower sides of the photo-peak window. SPECT data acquisition was completed in 45 frames with four cycles of 210 s/cycle scanning over a 180° acquisition range in 4° steps. After SPECT data acquisition, a CT scan was performed for attenuation correction. SPECT images were reconstructed by an iterative algorithm using the three-dimensional ordered subset expectation maximization (3D-OSEM) method with eight iterations, 10 subsets, and a 6-mm Gaussian filter. CT attenuation correction with SC (ACSC) and without SC (CTAC) were applied for SPECT reconstruction.

### 2.3. SBR Calculation

The TB method was proposed to simplify the SBR calculation and to reduce the effects of partial volume artefacts [22]. To improve the reproducibility and to avoid operator-dependent variation, a semi-automatic count-based method was proposed, where the VOI_st_ was determined automatically in a trapezoidal VOI (VOI_t_) drawn manually in the basal ganglia region including the whole striata [18]. In order to avoid extra-striatal heterogeneous tissue counts, the average striatum volume of 11.2 mL was applied to determine VOI_st_. Details of the semi-automatic method are described elsewhere [18]. In brief, striatum-containing slices were detected by visual observation, and the VOI_t_ completely covering the bilateral striata was applied manually on the average SPECT image. The 1052 most intense voxels identical to the average striatum volume were extracted automatically from the VOI_t_. Next, the VOI_ref_ was drawn manually in the occipital region, avoiding the CSF and sinuses. Finally, SBR was calculated from the mean counts of the VOI_st_ and VOI_ref_ using the following equation:

$$\mathrm{SBR}=\frac{\left[\left({\mathrm{VOI}}_{\mathrm{st}}\;\mathrm{mean}\;\mathrm{count}\right)-\left({\mathrm{VOI}}_{\mathrm{ref}}\;\mathrm{mean}\;\mathrm{count}\right)\right]\;\left(/\mathrm{mL}\right)}{[{\mathrm{VOI}}_{\mathrm{ref}}\;\mathrm{mean}\;\mathrm{count}]\;\left(/\mathrm{mL}\right)}$$

For fully automatic operation, an average image of all slices was firstly created from the original SPECT data. Extra-cranial noisy voxels were then excluded from the average image in several steps. Firstly, noisy clusters outside the skull in a size of 1000 voxels or smaller were removed from the binary data of the average image by creating a mask image. Distant voxel clusters outside the skull were removed by applying the mask to all slices of the SPECT image. The voxel clusters adjacent to the skull were excluded by applying a low voxel value less than one, which successfully removed the relatively low-intensive voxels. A filter mask image to remove clusters outside the skull less than 500 voxels was applied again to all slices, and the final SPECT image without any active voxels outside the head was obtained.

**Figure 1:**
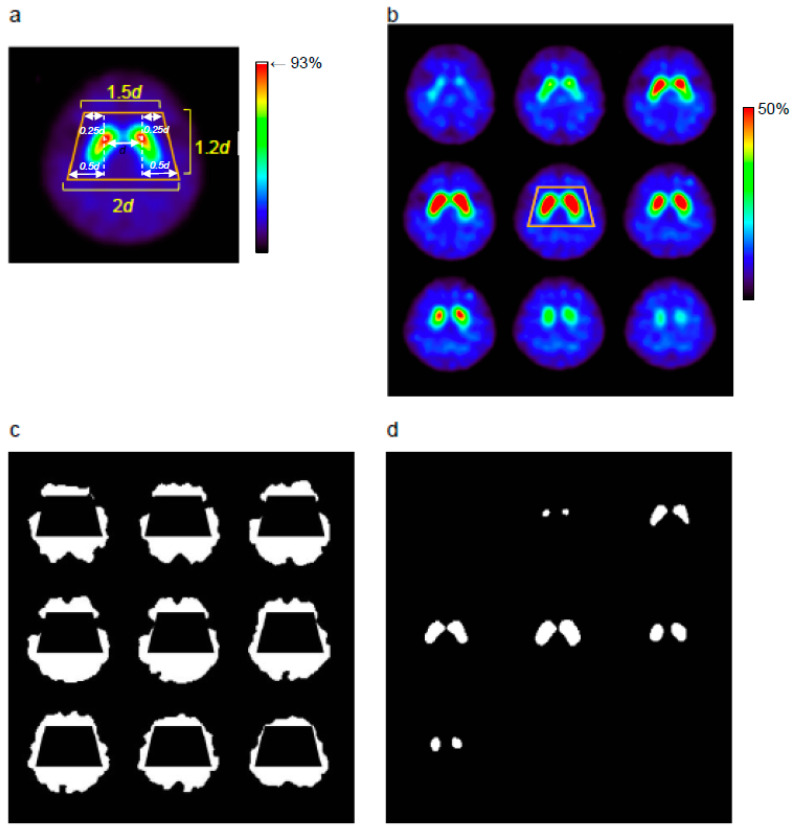
The trapezoidal volume of interest (VOI_t_) was created automatically in the striatal region based on the maximum count voxels of the striata (**a**). The white color (>93%) in the right caudate head indicates the peak count voxel in the right striatum, and the white cluster in the left caudate head includes the maximum voxel of the left side. The size of VOI_t_ was determined by the distance (*d*) of the most intense voxels of the bilateral striata. The VOI for the reference region (VOI_ref_) was determined from the whole brain region (**b**) after excluding low-count voxels of cerebrospinal fluid (CSF) space and VOI_t_ (**c**). Finally, a bilateral VOI of the striatum (VOI_st_) mask was created automatically by extracting the most intense 1052 voxels (= 11.2 mL) on each side [18] (**d**).

VOI_t_ was then created automatically based on the maximum voxel within the striatal region on the average image created above. The average brain image was split into separate volumes of right and left hemispheres at the midline of matrix size, i.e., 1–64 on horizontal axis for the right hemisphere and 65–128 for the left. Because the brain was located at the center of the field of view (FOV) in the step of SPECT image reconstruction, the left and right striata were always included in each hemisphere separately, especially at the peak count. Since the caudate head usually shows relatively greater uptake than any other regions even in cases of low striatal uptake [15], the four vertices of VOI_t_ were determined from the bilateral maximum points. Using the distance (*d*) of the bilateral maximum voxels in the striata, VOI_t_ was created as a trapezoid of 1.5*d* (= 0.25*d* × 2 + *d*) in top (anterior side), 2*d* in bottom (posterior side), and 1.23*d* height (Figure 1a). Each side vertex was located at the same distance from the bilateral maximum voxels, which provided a symmetric VOI_t_ with the mid-point of the bilateral maximum voxels located at the center of the trapezoid. Finally, the largest consecutive slices including the bilateral striatal clusters were selected and two additional slices were also included for the slice range of VOI_t_ to avoid a lack of striatal voxels. The VOI_t_ was applied throughout the total striatal slice range. The VOI_st_ was then determined automatically inside the VOI_t_ using the same procedure as the semi-automatic method described above.

The VOI_ref_ was determined from the remaining part of the brain after VOI_t_ setting (Figure 1B). The VOI_ref_ mask image was created using various thresholds determined from the maximum VOI_ref_ count to exclude low-count voxels, followed by the average VOI_ref_ count calculation after application of the mask image. The programs of both semi- and fully automatic methods were created on Matlab R2014 (Mathworks, Natick, MA, USA).

### 2.4. Statistical Analysis

In order to determine the most appropriate threshold of VOI_ref_ cut-off level in the automatic method, we calculated SBR for the previous 200 patients’ data using several thresholds and compared them with the results of the semi-automatic method. SBR values from the semi- and the fully automatic methods with the appropriate threshold were compared for the previous 200 and the present 105 patients’ data separately to assess the diagnostic accuracy for PS. The SBR values of the dominant side for the PS group and the mean of both sides for the non-PS group were used for statistical comparison. Receiver operating characteristic (ROC) analysis was applied, and the areas under the curves (AUCs) were compared between the methods and between the different image reconstructions [23,24]. The SBR values obtained by the new method were also compared with those from the TB method (SBR_Bolt_) [22] obtained using a specific software program (DaT View, AZE Inc., Tokyo, Japan). Repeated-measures analysis of variance (ANOVA) was applied for comparison of various SBR values followed by the paired *t*-test as a post hoc test. Pearson’s correlation analysis was used for evaluation of linear regression. The statistical significance was evaluated using Medcalc (ver. 19.1.5, Medcalc Software Ltd., Ostend, Belgium), and *p*-values less than 0.05 were considered significant.

## 3. Results

The mean time for SBR calculation of each subject was 9 ± 1 s, which was significantly faster than 2–3 min on average for the semi-automatic method. The slice range of VOI_t_ was confirmed in all cases by visual observation for the striatum-containing slices, and no errors were found in the new patient images studied.

**Figure 2:**
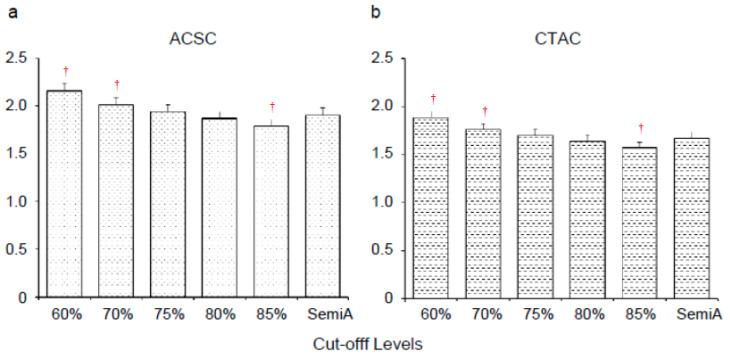
Specific binding ratio (SBR) values (mean ± standard error (SE)) obtained from our previous data of 200 patients with various VOI_ref_ thresholds. Single-photon emission computed tomography (SPECT) images were reconstructed by two methods of attenuation correction with and without scatter correction (ACSC (**a**) and CTAC (**b**)). Cut-off levels of 75% and 80% of the maximum VOI_ref_ count showed no significant difference from the results calculated using the semi-automatic method (semi-A). ^†^ *p* < 0.0001.

Figure 2 shows the average SBR values of our previous 200 patients obtained from the fully automatic method with various thresholds for VOI_ref_. Cut-off levels of 75% and 80% of the maximum VOI_ref_ count showed no significant difference from the semi-automatic method in images of either ACSC (Figure 2a, 75%: 1.94 ± 0.08 (*p* = 0.42) and 80%: 1.87 ± 0.07 (*p* = 0.22) for fully automatic vs. 1.91 ± 0.08 for semi-automatic method) or CTAC (Figure 2b, 75%: 1.70 ± 0.06 (*p* = 0.19) and 80%: 1.64 ± 0.06 (*p* = 0.31) for fully automatic vs. 1.67 ± 0.06 for semi-automatic method). In the present study, the cut-off level of 75% of the maximum VOI_ref_ count was used for further SBR evaluation in the automatic method.

**Figure 3:**
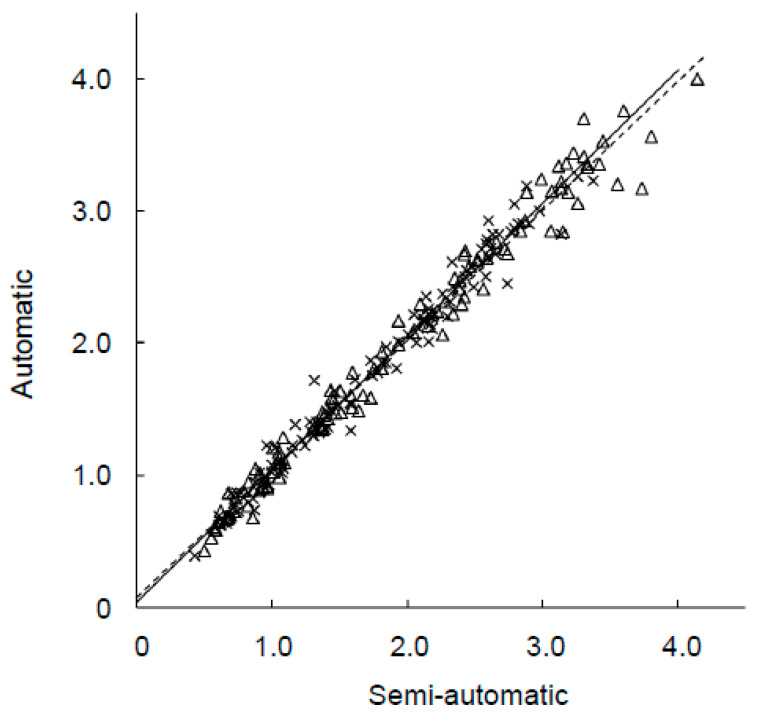
Scatter plot of SBR values from the semi- and fully automatic methods showed good linear correlations in both ACSC (Δ: *y* = 0.98*x* + 0.07, dashed line) and CTAC (×: *y* = 1.00*x* + 0.04, solid line) image data (*r* = 0.99).

**Figure 4:**
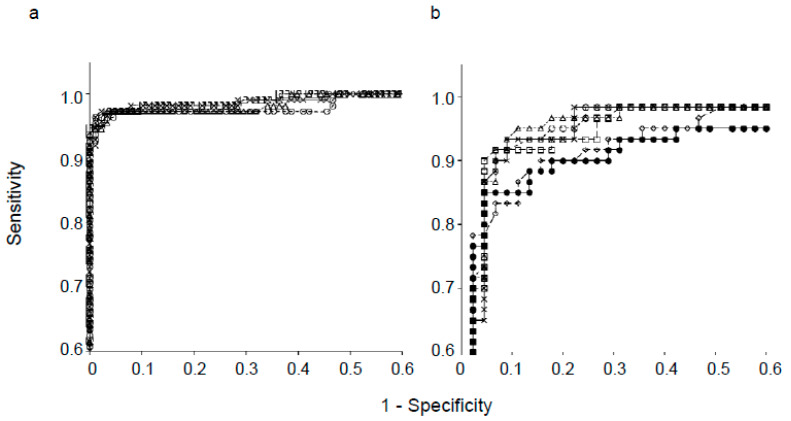
Receiver operating characteristic (ROC) analysis showing no significant difference in diagnostic accuracy between the semi- and fully automatic methods. (**a**) ROC of previous patient data (*N* = 200); area under the curve (AUC) = 0.992 (×) vs. 0.987 (○) with ACSC (*p* = 0.24) and 0.993 (☐) vs. 0.989 (△) with CTAC (*p* = 0. 37) for the semi- and fully automatic methods, respectively. (**b**) ROC of new patient data for the semi-, fully automatic, and Tossici–Bolt (TB) methods (*N* = 105); AUC = 0.965 (×) vs. 0.968 (○) (*p* = 0.37) with ACSC and 0.963 (☐) vs. 0.968 (△) (*p* = 0.50) with CTAC for semi- and fully automatic methods, respectively. AUCs of the TB method are 0.925 for ACSC (◇) and 0.917 for CTAC (●), significantly inferior to our methods (*p* = 0.035 and 0.033, respectively).

Figure 3 shows good linear correlations in SBR values between the semi- and fully automatic methods for both ACSC and CTAC images (*r* = 0.99). ROC analysis showed no significant difference in diagnostic accuracy between the semi- and fully automatic methods for both our previous (Figure 4a) and the present patient groups (Figure 4b). AUCs of the semi- and fully automatic methods for the previous 200 patients were 0.992 vs. 0.987 (*p* = 0.24) in ACSC and 0.993 vs. 0.989 in CTAC (*p* = 0.37). In the new 105 patients, the AUCs of ACSC and CTAC were 0.965 and 0.963 vs. 0.968 for the semi- and fully automatic methods and did not show a significant difference (*p* = 0.37 and 0.50, respectively). AUCs of the TB method in the present patient data were 0.925 for ACSC and 0.917 for CTAC, which were inferior to those of the fully automatic method (*p* < 0.05) (Figure 4b).

**Figure 5:**
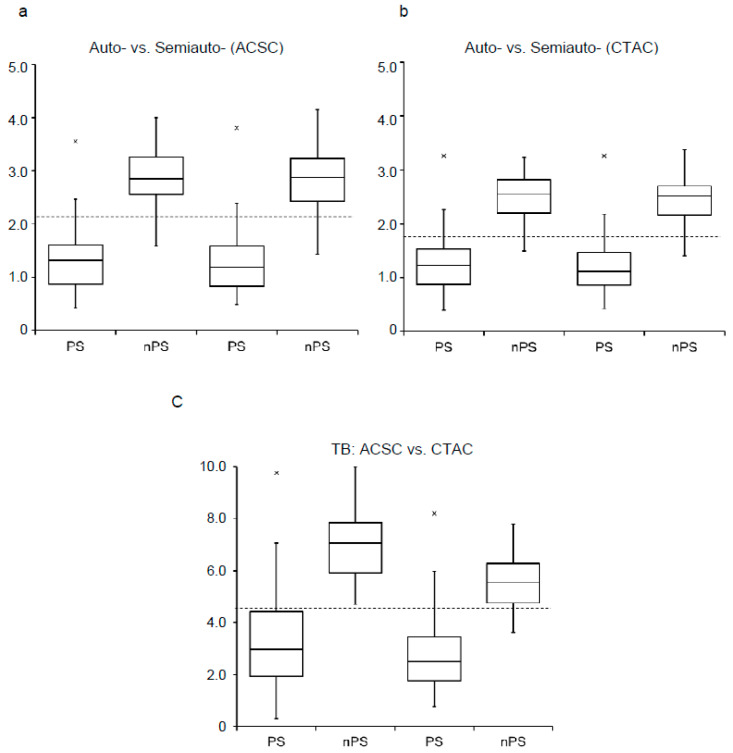
The parkinsonian syndrome (PS) group showed significantly greater SBR compared with the non-PS group (nPS) in the semi-and fully automatic methods for both ACSC (**a**) and CTAC (**b**) images (*p* < 0.0001). The same cut-off values (ACSC = 2.15, CTAC = 1.88) differentiated the PS and non-PS groups with excellent diagnostic accuracy. The TB method with 4.5 cut-off (**c**) showed fair accuracy (see Table 1).

The fully automatic method differentiated the PS group from the non-PS group well in both ACSC and CTAC images (*p* < 0.0001) (Figure 5). Taking an SBR cut-off value of 2.15 for ACSC and 1.88 for CTAC, determined by ROC analysis, both the semi- and fully automatic methods showed similar sensitivity and specificity. Table 1 summarizes the sensitivity, specificity, positive and negative predictive values, and diagnostic accuracy of the two methods for our previous and new patient groups. In the TB method, the SBR_Bolt_ cut-off value of 4.5 with CTAC images, which was reported previously [22], showed lower sensitivity and specificity compared with our methods (Table 1).

## 4. Discussion

We proposed a fully automatic method for SBR calculation of [^123^I]FP-CIT SPECT in the present study to avoid operator-induced variability and to improve reproducibility. The reproducibility of the new method with two sets of patient data was identical to the previous semi-automatic method in diagnostic accuracy based on the ROC analysis. The handling time was significantly improved to 9 ± 1 s/image for each SBR calculation compared with 2–3 min/image for the semi-automatic method. In DaT-SPECT imaging, accuracy and reproducibility are equally important for patient follow-up, as well as for multicenter studies. The proposed method simplified SBR calculation without application of any template or any other manual handling steps, providing excellent diagnostic accuracy and reproducibility.

In several previous studies, a template was created from normal healthy individuals and used for detection of the VOI of the striata, which made the procedure complicated and time-consuming [16,25]. The template-based automatic normalization method may induce errors due to misregistration and partial volume effects caused by atrophic changes or other pathologic or physiologic factors. Thus, the reliability of the results depends on the accuracy of image registration into the predefined template. In our previous semi-automatic method, VOI_t_ was drawn manually just to detect the striatal position [18]. In the new automatic method, VOI_t_ was created based on the maximum count in the striatal region without any manual steps. The VOI_st_ in our method was determined based on the maximum count in the VOI_t_ without using any predefined templates. Non-specific tracer binding in the background of the brain may cause VOI_t_ and VOI_st_ setting errors in cases of low striatal uptake. However, the fullyautomatic method successfully created the VOI_t_, including the bilateral striata, even in cases of very low striatal uptake, similarly to the semi-automatic method with manual VOI_t_ setting. There were no patients with VOI_t_ outside of the striatal region in the present cases.

Since the variability of the VOI_ref_ count concentration affects the SBR values significantly [19], the selection of an appropriate reference region is very important for precise SBR calculation. SBR was calculated using the specific reference region such as the occipital lobes or the whole brain VOI excluding the CSF space, but including both the gray and white matter, although they showed good diagnostic accuracy [26]. The gray and white matter have different pharmacokinetic dynamics in terms of radiotracer uptake [20,27], and the non-specific count should be calculated from the gray matter avoiding the CSF space and white matter to obtain SBR more precisely. Similar non-specific binding is expected between the reference and the striatal regions. However, background counts in the [^123^I]FP-CIT SPECT images were similar in both gray and white matter, and strict discrimination of these may not be so important in terms of the reliability of results. On the other hand, a large reference region is supposed to reduce the variability of VOI_ref_ counts by minimizing the variation of anatomical VOI position [17]. In the present study, the threshold for VOI_ref_ was determined by firstly comparing various percentages of the threshold to exclude effects of CSF and low counts in the white matter. To determine a suitable threshold, we used a relatively large dataset from our previous study [18]. The mean SBR values decreased gradually according to the cut-off level for exclusion of low-intensive voxels in the reference region, as expected (Figure 2). We found that the cut-off percentages of 75% and 80% of the maximum count showed no significant difference from our previous semi-automatic method, which was consistent with a previous report by Buchert et al. [19]. Thus, we selected 75% as the cut-off threshold, which was the same percentage as their result.

The performance of the proposed automatic method was assessed by the accuracy, reproducibility, and usability of the method [28]. For assessment of the accuracy, we compared the results of the automatic method with our previously developed semi-automatic method, as well as with the SBR_Bolt_ of the TB method, commonly used in Japan. The new automatic method showed high similarity compared with our semi-automatic method and better diagnostic accuracy than the TB method for both ACSC and CTAC images, because our method is not affected by noisy voxel counts outside of the striata [18]. Thus, the method seems to have advantages even compared with the automatic methods previously reported [15,16], because it does not require templates, registration steps, and setting parameters for striatal VOIs. Since our new method is fully automated, reproducibility is warranted except for the cases of significantly low tracer accumulation in the striatum, although we did not find any cases with errors in the present 105 patients. The diagnosis of DaT-SPECT would not alter in those cases of very low striatal counts causing incorrect VOI_t_ and VOI_st_ location because the striatal uptake should be the background level [18]. The usability was improved in the new method, with reduction of the SBR calculation time and no reliance on handling skills. Our automatic method is user-friendly with excellent accuracy and reproducibility without any training for running the program in cases of clinical assessment of neurodegenerative diseases using [^123^I]FP-CIT SPECT images.

## 5. Conclusions

Our proposed method calculated SBR automatically in a very short time with excellent diagnostic accuracy, which is essential to obtain quantitative results and ideal reproducibility.

## Figures and Tables

**Table 1:** Sensitivity, specificity, positive predictive value (PPV), negative predictive value (NPV), and accuracy of semi-automatic and fully automatic methods.

SPECT Data	Method	Patient Group	Sensitivity (%)	Specificity (%)	PPV (%)	NPV (%)	Accuracy (%)
ACSC	Semi-automatic	Previous	97.3	93.2	94.8	96.5	97.3
		New	93.3	93.2	94.9	91.1	93.3
	Automatic	Previous	97.3	95.5	96.5	96.6	97.3
		New	91.7	95.5	96.5	89.4	91.7
CTAC	Semi-automatic	Previous	97.3	95.5	96.5	96.6	97.3
		New	91.7	95.5	96.5	89.4	91.7
	Automatic	Previous	97.3	95.3	96.5	96.6	97.3
		New	91.7	95.5	96.5	89.4	91.7
	TB	New	88.3	82.2	86.9	84.1	88.3

ACSC: CT attenuation correction with scatter correction (SC), CTAC: CT attenuation correction without SC, PPV: positive predictive value, NPV: negative predictive value, TB: Tossici–Bolt method [22].
